# Posterior and Anterior Spinal Fusion for the Management of Deformities in Patients with Parkinson's Disease

**DOI:** 10.1155/2013/140916

**Published:** 2013-09-02

**Authors:** Masashi Sato, Takeshi Sainoh, Sumihisa Orita, Kazuyo Yamauchi, Yasuchika Aoki, Tetsuhiro Ishikawa, Masayuki Miyagi, Hiroto Kamoda, Miyako Suzuki, Gou Kubota, Yoshihiro Sakuma, Kazuhide Inage, Yasuhiro Oikawa, Junichi Nakamura, Masashi Takaso, Gen Inoue, Tomoaki Toyone, Kazuhisa Takahashi, Seiji Ohtori

**Affiliations:** ^1^Department of Orthopaedic Surgery, Graduate School of Medicine, Chiba University, 1-8-1 Inohana, Chuo-ku, Chiba 260-8670, Japan; ^2^Department of Orthopaedic Surgery, Toho University Sakura Medical Center, Chiba 285-8741, Japan; ^3^Department of Orthopaedic Surgery, Sanmu Medical Center, Chiba 289-1326, Japan; ^4^Department of Orthopaedic Surgery, Chiba Cancer Center, Chiba 260-8717, Japan; ^5^Department of Orthopaedic Surgery, Kitasato University, Tokyo 252-0375, Japan; ^6^Department of Orthopaedic Surgery, Teikyo University Chiba Medical Center, Chiba 299-0111, Japan

## Abstract

*Introduction*. Spinal scoliosis and kyphosis in elderly people sometimes cause severe low back pain. Surgical methods such as osteotomy are useful for correcting the deformity. However, complications during and after surgery are associated with the osteotomy procedure. In particular, it is difficult to manage deformity correction surgery for patients with Parkinson's disease. Here, we present two cases of combined anterior and posterior surgery for deformity in patients with adult scoliosis and kyphosis due to Parkinson's disease. *Case Presentation*. Two 70-year-old women had spinal scoliosis and kyphosis due to Parkinson's disease. They had severe low back pain, and conservative treatment was not effective for the pain. Surgery was planned to correct the deformity in both patients. We performed combined posterior and anterior correction surgery. At first, posterior fusions were performed from T4 to the ilium using pedicle screws. Next, cages and autograft from the iliac crest were used in anterior lumbar surgery. The patients became symptom free after surgery. Bony fusion was observed 12 months after surgery. *Conclusions*. Combined posterior and anterior fusion surgery is effective for patients who show scoliosis and kyphosis deformity, and symptomatic low back pain due to Parkinson's disease.

## 1. Introduction

Spinal kyphosis in elderly people sometimes causes serious problems. It occurs as a consequence of the loss of the physiological lordotic curve due to senile kyphosis, which is attributed to osteoporosis, disk degeneration, and impairment of back muscles [1, 2]. The postural abnormality associated with kyphosis can cause chronic low back pain, and in later stages it can also disturb standing and gait by affecting balance [3]. Kyphoscoliosis due to Parkinson's disease (PD) has been reported since 1999 [4]. It is estimated that about 7% of patients with PD have kyphoscoliosis [5]. The frequency of kyphoscoliosis in the population of PD patients is higher than that of scoliosis in an age-matched population without PD [6].

There have been multiple surgical procedures described for the treatment of spinal kyphotic deformity, including Smith-Petersen osteotomy (SPO), pedicle subtraction osteotomy (PSO), and vertebral column resection (VCR) procedures. The technique used to correct spinal kyphotic deformity depends on factors such as the severity of the deformity, the flexibility of the deformity, and whether the kyphosis is more of a rounded, long sweeping kyphosis or a short, angular one [7, 8]. Suk et al. reviewed the treatment of 70 patients with spinal deformity using posterior VCR, and complications were encountered in 34% of patients [9]. Kim et al. reported the treatment of 35 consecutive patients with sagittal imbalance using lumbar PSO, and there was an occurrence of pseudarthrosis and need for revision surgery in 23% of patients [10]. Spinal surgery in PD is an extremely demanding procedure, known for its high complication rate and the frequent need for surgical revision [11]. There is currently no consensus about the management of major spinal deformities in PD. In this regard, we need to be aware of the high complication rate and the need for revision surgery because of complications after these surgical methods in patients with PD.

Here, we present two cases of combined anterior and posterior surgery in patients with kyphoscoliosis deformity due to PD.

## 2. Cases Presentation

### 2.1. Case 1

**Figure 1:**
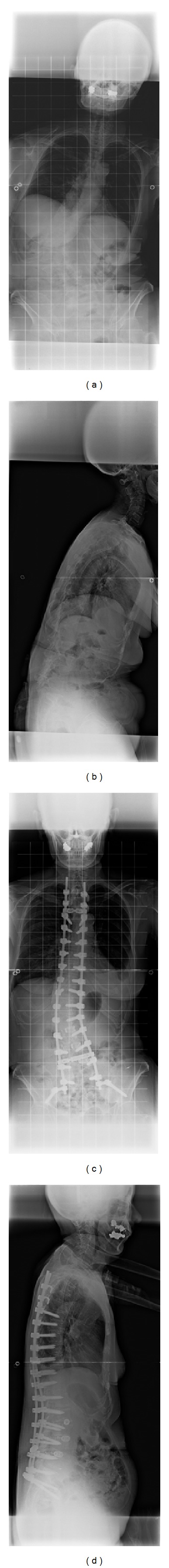
X-ray film images from a 70-year-old woman before and after surgery (Case 1). Anterior-posterior view (a) and lateral view (b) before surgery and anterior-posterior view (c) and lateral view (d) after surgery.

**Figure 2:**
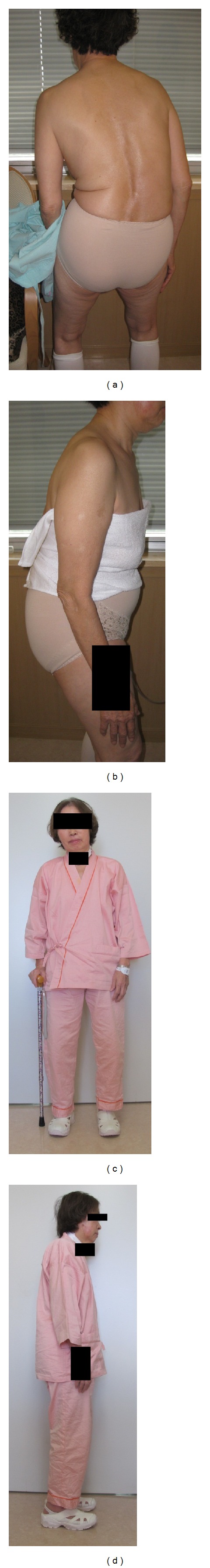
Posture before surgery and after surgery in Case 1. Posterior-anterior view (a) and lateral view (b) before surgery and anterior-posterior view (c) and lateral view (d) after surgery.

Written signed consent was received from the patient before treatment. In June 2011, a 70-year-old woman with PD presented with a 20-year history of low back pain. Visual analogue scale (VAS) of low back pain was 9 (worst 10). Motor weakness using manual muscle testing (MMT) was not observed, and sensory examination using pin prick test confirmed no abnormality. Deep tendon reflex was normal in both legs. There was apparent urinary disturbance. Bilateral straight leg raising test results were negative. Posture and X-ray image examinations showed spinal scoliosis and kyphosis (Figures 1 and 2). Because conservative treatment was not effective, surgery was planned. We performed combined posterior and anterior fusion surgery. First, posterior correction and fusion were performed from T4 to the ilium using pedicle screws. We did not perform an osteotomy or remove facet joints. Next, anterior interbody fusion using cages and autograft from the iliac crest was performed from L2-3 to L4-5 (Figure 1). At 12 months after surgery, bone fusion was observed, and there was no breakage of instrumentation. Sagittal and coronal posture, low back pain using VAS (from 9 to 2), and balance parameters (see in Table 1) improved after surgery compared with before surgery (Figures 1 and 2 and Table 1).

### 2.2. Case 2

Written signed consent was received from the patient before treatment. In April 2012, a 70-year-old woman with PD presented with a 15-year history of severe low back pain. VAS of low back pain was 9 (worst 10). She could not walk without a crutch because of this back problem. There was apparent urinary disturbance, motor weakness, and sensory disturbance using MMT and pinprick test.

**Figure 3:**
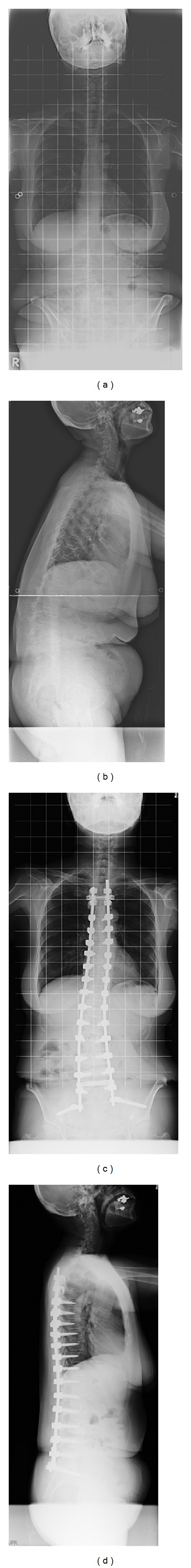
X-ray film images from a 70-year-old woman before and after surgery (Case 2). Anterior-posterior view (a) and lateral view (b) before surgery and anterior-posterior view (c) and lateral view (d) after surgery.

**Figure 4:**
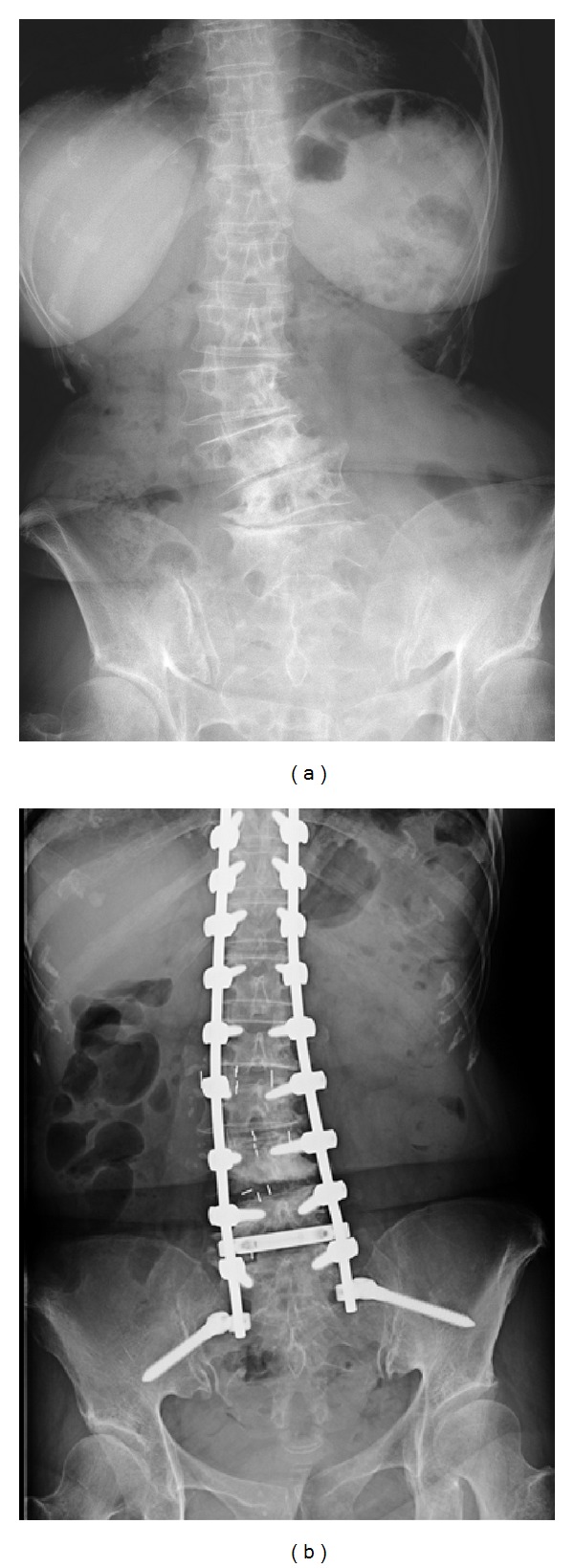
Magnification of X-ray film images of lumbar spine before and after surgery in Case 2. Anterior-posterior view (a) before surgery, and anterior-posterior view (b) after surgery.

**Figure 5:**
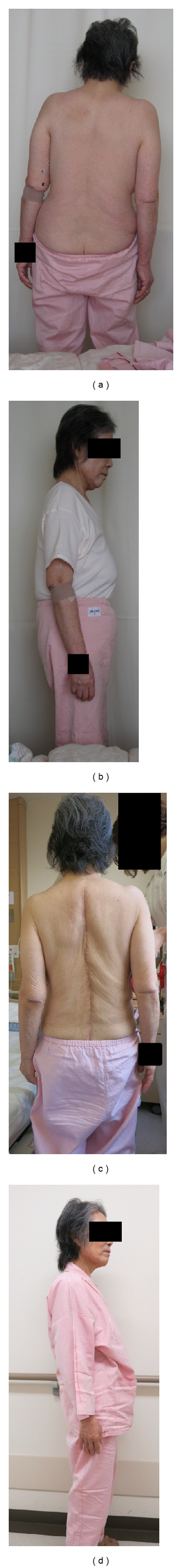
Posture before surgery and after surgery in Case 2. Posterior-anterior view (a) and lateral view (b) before surgery, and posterior-anterior view (c) and lateral view (d) after surgery.

X-ray image examination showed spinal scoliosis and kyphosis (Figure 3). We performed combined posterior and anterior fusion surgery for her deformity. First, posterior correction and fusion was performed from T4 to the ilium using pedicle screws and transforaminal interbody fusion at the L5-S1 level. We did not perform osteotomy. Next, direct lateral interbody fusion using cages and autograft from the iliac crest was performed at the L3-4 and L4-5 levels (Figures 3 and 4). At 12 months after surgery, bone fusion was observed, and there was no breakage of instrumentation. Sagittal and coronal posture, low back pain using VAS (from 9 to 1), and balance parameters (see in Table 2) improved after surgery compared with before surgery (Figures 3 and 5 and Table 2).

## 3. Discussion

In the current report, we present two patients with PD and adult kyphosis. We selected a combination of anterior and posterior fusion surgery to correct the deformity in the patients. Posture, low back pain, and balance parameters improved after surgery compared with before surgery, and we concluded that this combination was effective for kyphoscoliosis in patients with PD.

Osteotomy is effective for correction of kyphosis. However, high rates of complications have been reported. The Scoliosis Research Society Morbidity and Mortality Committee reported the short-term complication rate in patients undergoing treatment of thoracolumbar fixed sagittal plane deformity [12]. Five hundred seventy-eight cases were analyzed; the rate of complication was 29.4% in all patients, and there were deaths (0.5%). The most common complications were durotomy (5.9%), wound infection (3.8%), new neurologic deficit (3.8%), implant failure (1.7%), wound hematoma (1.6%), epidural hematoma (1.4%), and pulmonary embolism (1.0%). Procedures including osteotomy had a higher complication rate (34.8%) than cases not including an osteotomy [12].

PD patients with osteoporotic bones face the loss of function of the spinal extensor muscles directly related to this disease and to age-associated fatty degeneration [11]. By contrast, patients with camptocormia were compared to age-matched patients without camptocormia [13]. In patients with camptocormia, computed tomographic scans and magnetic resonance imaging showed heterogeneous appearance of the spinal muscles with areas of low density [13]. The main microscopic change in camptocormia was the increase of fibrous tissue and atrophy, frequently with a lobular pattern [13]. There is the possibility of total back muscle atrophy in PD patients, similar to patients with camptocormia, and this muscle atrophy may induce spinal deformity in patients with PD. In this regard, long posterior fixation may be necessary to maintain correction for deformity of PD patients.

Neuromuscular disease complicates spinal surgery at any age. Banta et al. reviewed 30 papers on the treatment of neuromuscular scoliosis (902 pediatric patients with myelomeningocele, muscular dystrophy, cerebral palsy, and spinal muscular atrophy) [14]. Most patients had an arthrodesis with posterior instrumentation. Pseudarthroses and instrumentation failure were extremely common, and proximal junctional kyphosis was reported frequently; revision surgery was sometimes needed [14]. Babat et al. reported difficulty of spinal surgery in patients with PD [11]. Overall, 86% of patients required additional revision surgery [11]. The reason for revision surgery was instability at the same or an adjacent level, hardware failure or pullout, instability at a remote spinal segment, and development of wound infections during the course of treatment [11].

For treatment of deformity due to PD, a few authors recently have reported posterior long fusion. However, some complications have also been reported. Wadia et al. reported surgical correction of kyphosis in two patients with camptocormia due to PD [15]. The first stage was to place instrumentation from T3 to the pelvis, and the second stage was to achieve deformity correction via an L3 pedicle subtraction osteotomy in 2 patients [15]. Symptoms improved after surgery; however, at 24 months after surgery, the rod had fractured below the L5 vertebral body in one patient, suggestive of a pseudarthrosis [15]. Koller et al. performed a retrospective review of 23 PD patients treated surgically for spinal disorders [16]. Most of the patients required fusion to S1, S2, or the ilium, but surgical complications were noted in patients without fusion to S1, S2, or the ilium [16]. They concluded that because PD patients have a weak muscular posterior tension band, a generally flexed and stooped posture increases with disease severity, resulting in unfavorable biomechanics of a long lever arm at the lumbosacral junction [16]. Hence, using additional sacral fixation points or iliac screw fixation was needed [16]. Bourghli et al. treated 12 PD patients with major spinal deformities using a posterior-only approach for spinal fusion from T2 to the sacrum [17]. This study indicates that significant correction of sagittal and frontal balance enables good clinical and radiologic results [17]. However, there were 3 cases of material failure in patients who had undergone pedicle subtraction osteotomy and the failure occurred in the 3 patients at the osteotomy level with bilateral rod fracture [17]. These studies suggest that posterior long fusion is necessary for correction (upper thoracic level to ilium), but that osteotomy is sometimes a cause of implant failure.

In the current study, we used both anterior and posterior surgery without osteotomy. Generally, the thoracic spine is more stable compared with the thoracolumbar and lumbar spine. Berven et al. reviewed the long-term results of posterior-only surgery for the treatment of instability in the thoracic spine, and single-stage posterior surgery was sufficient for extreme instability of the spine [18]. By contrast, reviews of repeated fusion for failed surgery in the lumbar spine show a lower fusion rate, and most techniques reported in the literature concentrate only on posterior fusion [19, 20]. By contrast, Albert et al. attempted to determine the radiographical results of a combined anterior-posterior surgical procedure to manage instability of the lumbosacral spine and concluded that results are superior to those previously reported for the use of a posterior technique alone [21]. A biomechanical study revealed the importance of the anterior portion of the spine for spinal stability. The role of intervertebral discs in degenerative spinal instability has previously been clarified by means of biomechanical cadaver studies [22, 23]. Another cadaver study revealed that axial rotational motion was more affected by disc degeneration rather than posterior facet joints, and segmental motion increased with increasing severity of disc degeneration [22]. For these reasons, we selected second anterior surgery for 2 PD patients because the PD patients showed an unstable lumbar spine.

The current study is limited by the number of patients presented. Nevertheless, further studies into the efficacy of combined posterior and anterior fusion surgery for patients who show kyphoscoliosis deformity and symptomatic low back pain due to PD are warranted.

In conclusion, combined posterior and anterior fusion surgery was effective for stabilizing the spine in patients who showed scoliosis and kyphosis due to PD. The patients became symptom-free after surgery, and good sagittal and coronal balance was achieved.

## Figures and Tables

**Table 1:** Radiographic parameter (Case 1).

	Before surgery	After surgery
C7-plumb (SVA) (mm)	193	56
Thoracic kyphosis (TK) (deg.)	24.4	29.1
Lumbar lordosis (LL) (deg.)	−2.1 (kyphosis)	29.6
		
Pelvic tilt (PT) (deg.)	44.5	30
Pelvic incidence (PI) (deg.)	56.7	53.3
Sacral slope (SS) (deg.)	14.3	29.6
		
Cobb angle (deg.) (L1 to L4)	48.2	20.1
C7-plumb (frontal) (mm)	41	13

**Table 2:** Radiographic parameter (Case 2).

	Before surgery	After surgery
C7-plumb (SVA) (mm)	148	51
Thoracic kyphosis (TK) (deg.)	6.3	22.4
Lumbar lordosis (LL) (deg.)	−18.2 (kyphosis)	13.6
		
Pelvic tilt (PT) (deg.)	43.7	24.8
Pelvic incidence (PI) (deg.)	50.7	45.1
Sacral slope (SS) (deg.)	5.4	16.8
		
Cobb angle (deg.) (L1 to L4)	30.4	12.8
C7-plumb (frontal) (mm)	21	18
